# Molecular Phylogeny and Phylogeography of *Potentilla multifida* L. agg. (Rosaceae) in Northern Eurasia with Special Focus on Two Rare and Critically Endangered Endemic Species, *P. volgarica* and *P. eversmanniana*

**DOI:** 10.3390/plants9121798

**Published:** 2020-12-18

**Authors:** Ivan A. Schanzer, Alina V. Fedorova, Olga V. Shelepova, Guzyaliya F. Suleymanova

**Affiliations:** 1 Tsitsin Main Botanical Garden of Russian Academy of Sciences, Botanicheskaya Str., 4, 127276 Moscow, Russia; alina_77777@mail.ru (A.V.F.); shov_gbsad@mail.ru (O.V.S.); 2 Khvalynsky National Park, Oktyabrskaya Str., 2B, 412787 Khvalynsk, Russia; suleymanovagf@mail.ru; 3 Saratov State University, Astrakhanskaya Str., 83, 410012 Saratov, Russia

**Keywords:** *Potentilla* sect. *Multifida*, *Potentilla volgarica*, *Potentilla eversmanniana*, *Potentilla multifida* agg., *ndhC-trnV*, *psbA-trnH*, ITS, genetic polymorphism, plastid haplotype genealogy

## Abstract

The results of a molecular genetic study of *Potentilla multifida* agg. using two plastid markers (*ndhC-trnV* and *psbA-trnH*) and a nuclear ITS marker suggested that this group comprises a number of relatively young and incompletely differentiated species widely distributed in Northern Eurasia. The sequences were analyzed using tree-based (maximum likelihood) and network-based (statistical parsimony network) approaches. The plastid data suggested incomplete lineage sorting, characteristic of the group as a whole. The nuclear ITS results demonstrated quite a different pattern, with mostly conspecific accessions shaping monophyletic clades. The majority of the *Potentilla* sect. *Multifidae* species studied possess few, usually closely related plastid haplotypes, or are even monomorphic. In contrast, *P. volgarica*, a narrow endemic from the Volga River valley, presents plastid haplotypes belonging to two distantly related groups. Such a pattern of genetic diversity in *P. volgarica* may be explained by a long persistence of the species within an extremely small distribution range, on the right bank of the Volga River, most likely representing a contemporary refugium. The genealogy of plastid markers in *P. volgarica* suggests that this species is ancestral to *P.* *eversmanniana,* another narrow endemic from the S Urals.

## 1. Introduction

The genus *Potentilla* L. comprises more than 300 species distributed worldwide in temperate areas and in mountainous regions in the tropics [1,2,3]. Hybridization, polyploidy, and apomixis are not rare among its species [4,5,6,7,8,9,10], which make the taxonomy of the group very complicated. The existing phylogenies of *Potentilla* and the Potentilleae tribe are based on relatively small subsets of taxa and are still far from comprehensive [2,11,12,13,14,15]. Even in the cases where the taxa sets of Potentilleae were quite comprehensive [2,13], some groups of *Potentilla* s. str. were underrepresented, including those that are the focus of our study. The taxonomy of the genus is, similarly, far from a definite assessment. Despite the existence of a number of relatively recent regional revisions and critical taxonomic accounts [7,16,17,18,19,20,21] the taxonomic system of the genus are still based on the monograph by Th. Wolf [1], and no comprehensive revision encompassing the whole genus has been done recently. Moreover, the very limits of the genus are subject to reconsideration in the light of recent phylogenetic studies that call into question the taxonomic rank of such groups as *Argentina* Hill, *Ivesia* Torr. & A. Gray, and *Horkelia* Cham. & Schltdl.

The genus includes many common widely distributed species along with geographically restricted endemics, some of which are thought to be extremely rare and close to extinction [22,23]. Though several species of *Potentilla* have been thoroughly studied already as to their population structure and phylogeography [24,25,26], such endangered endemics are not among them. At the same time, the very endemicity and rarity of some species may be questionable, because of the lack of clear-cut delimitation from morphologically similar, widely distributed species. *Potentilla volgarica* Juz. and *P. eversmanniana* Fisch. ex Ledeb. represent examples of such a situation. The former species is believed to be an endemic of the Middle Volga River valley in the vicinity of Khvalynsk, Saratov Province, Russia, with a distribution range of a few hundred square kilometers. In the IUCN Red List [22] it was listed as already extinct, but several new populations were later found in the same area [23]. *Potentilla eversmanniana* is known from a handful of isolated locations in the Southern Urals [7,23]. Its occurrence in Kazakhstan and Mongolia [7,27] is dubious. The IUCN Red List [22] listed it as vulnerable. Both species are included in the Red Data Book of the Russian Federation [23].

Furthermore, the two species are morphologically very similar to each other as well as to the widely distributed species *P. multifida* L. and *P. tergemina* Soják, all belonging to Wolf’s “grex” *Multifidae* [1], a group encompassing 27 species distributed through temperate and mountainous areas of Eurasia and North America. In modern works this group is usually assigned the rank of section [7,21]. None of its species was ever studied with molecular genetic methods, nor thoroughly analyzed for morphological variability except for genus or tribal level phylogenetic studies. The group is mostly represented by polyploids: *P. tergemina* (2n = 28, 33, 36), *P. multifida* (2n = 28, 42), *P. agrimonioides* M.Bieb. (2n = 42, 49–50), *P. anachoretica* Soják (2n = 28), *P. ornithopoda* Tausch (2n = 28, 42) [28], and *P. jenissejensis* Polozhij et W.A.Smirnova (2n = 4x, flow cytometric data) [29]. Many of these species remain taxonomically critical, and the group needs a thorough phylogenetic and taxonomic re-examination. The problem of species delimitation in the *P. multifida* group in general, and the assessment of the rank of *P. volgarica* and *P. eversmanniana*, in particular, are evident from a polemic on their taxonomy between two specialists in *Potentilla* taxonomy, late Professors R. Kamelin [7] and J. Soják [19]. Kamelin considered *P. volgarica* a “hybrid race of unknown origin”, and *P. eversmanniana* a “hybrid race (*P. multifida* × *P. conferta*) evidently more widely distributed in the past”. Soják completely rejected this view and described the two taxa as “clear and indisputable species”, differing morphologically primarily in the presence/absence of glandulous hairs on the upper leaf surface. The taxonomic treatment of the section *Multifidae* (Rydb.)A.Nelson is also controversial. Morphological differences of many species are vague, being essentially based on the number of quantitative characters of number of leaflet pairs, presence and abundance of glands on different parts of the plant, and shape and position of trichomes on the leaf undersurface and petioles. These characters are often dependent on the size and age of the plants. For example, *P. volgarica* differs from *P. eversmanniana* in the presence of glands on leaves and the less dense tomentose pubescence of the leaf undersurface [7,18]. These characteristics, however, vary considerably among plants in local populations of *P. volgarica*, so that at least some plants do not bear any glands on leaves at all. Moreover, *P. multifida* and *P. tergemina*, according to available keys, differ only by appressed vs. patent hairs on petioles, the latter character being typical for both *P. eversmanniana* and *P. volgarica*, albeit the hairs are longer than in *P. tergemina*. Hence, on morphological grounds, all the samples of *P. eversmanniana* kept at the Herbarium of Moscow University (MW) should be re-determined as *P. tergemina* having hairs shorter than 3 mm.

Trying to solve the problem of the taxonomic identity of *P. eversmanniana* and *P. volgarica*, we studied several populations of both species in the Bashkortostan Republic and Saratov Province of Russia, respectively, covering the whole range of *P. volgarica*. Herbarium collections kept at Moscow Lomonosov State University (MW), Tsitsin Main Botanical Garden (MHA), and Botanical Garden of Saratov State University (SARBG) were used to complement these data with specimens from other localities, not studied in the field, and samples of other morphologically similar species, chiefly characterized by deeply dissected leaflets, a key character of the section *Multifidae*, united by Soják [19] in his *P. multifida* agg. This enabled us to analyze a significant number of additional accessions.

The aims of the study were as follows:

1. To assess the genetic variability of *P. volgarica* and *P. eversmanniana* and to test whether they represent two separate species.
2. To assess the genetic distinctions of both species from *P. multifida* agg. sensu Soják [19] and other related species of the section *Multifidae*.
3. To assess the phylogenetic relationships of *P. multifida* agg. species and pinpoint the origin of disjunct isolated populations of *P. volgarica* and *P. eversmanniana* in the Russian Plain and the foothills of the Southern Urals, respectively.

## 2. Results

### 2.1. Plastid Data Analyses

The length of the *trnH-psbA* IGS varied from 376 bp to 488 bp in the ingroup (*Potentilla* sect. *Multifidae*) and from 308 bp to 416 bp in the outgroup (*Potentilla* species from other sections). The length of the *ndhC-trnV* IGS varied from 489 bp to 543 bp in the ingroup and from 506 bp to 585 bp in the outgroup. The length of the concatenated alignment was 1215 bp. The length of the alignment after trimming with the BMGE (Block Mapping and Gathering with Entropy) v. 1.1. software [30] and manually removing three ‘AT’ short repeats causing homoplasy in preliminary analyses was reduced to 910 bp. The final alignment contained 56 variable positions, of which 19 positions were parsimoniously informative, 37 positions were autapomorphic, and 64 sites were alignment gaps treated as missing data in further analyses.

**Figure 1:**
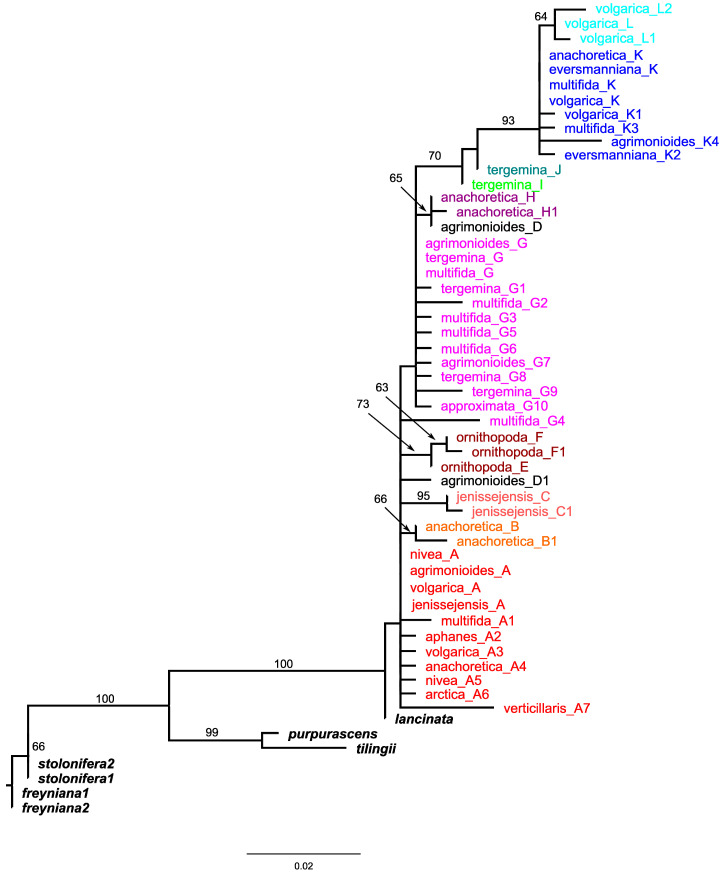
Maximum likelihood tree of *Potentilla multifida* agg. based on plastid genomic markers. Terminal names within the ingroup are followed by corresponding haplotype designations. Bootstrap support higher than 50% is indicated above branches. Different haplotype groups are highlighted with colors corresponding to those in Figure 2.

This trimmed alignment was used for a maximal likelihood analysis (Figure 1), which resulted in the inclusion of sequences of *P. multifida*, *P. tergemina*, *P. anachoretica*, *P. arctica* Lehm., *P. agrimonioides*, *P. aphanes*, *P. jenissejensis*, *P. ornithopoda*, *P. approximata* Bunge, *P. verticillaris* Stephan ex Willd., *P. volgarica*, *P. eversmanniana*, and *P. nivea* L. into the ingroup. The latter species initially was regarded as an outgroup member. The tree was poorly resolved and sequences of many samples were placed on short or zero length branches. That was indicative of incomplete lineage sorting within and among the taxa of *Potentilla* analyzed and justified the use of haplotype networks to reconstruct the relationships among them [31,32].

**Figure 2:**
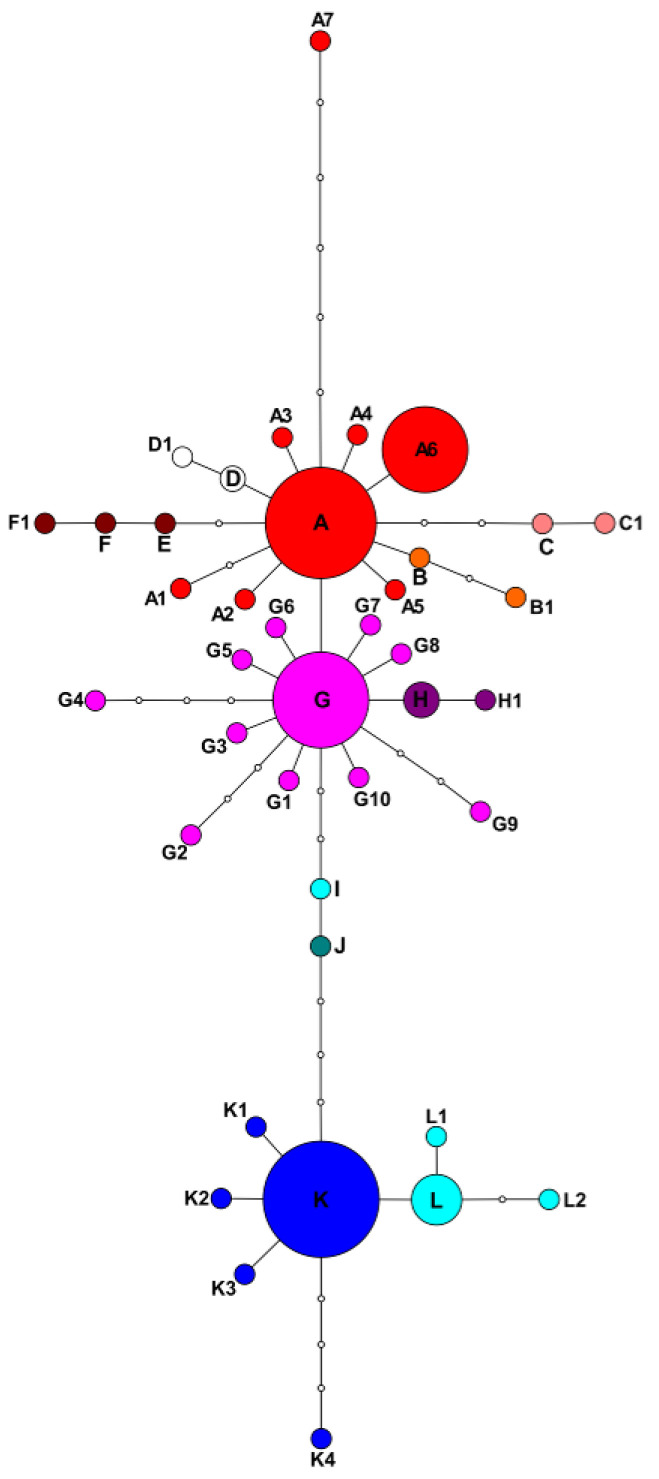
Statistical parsimony network of *Potentilla multifida* agg. plastid haplotypes. Bold letters designate internal haplotypes, letters and numbers designate tip haplotypes derived from corresponding internal ones. Colors distinguish haplotype lineages. Small empty circles indicate missing hypothetical haplotypes deduced by the program. Each edge corresponds to one mutational step.

**Figure 3:**
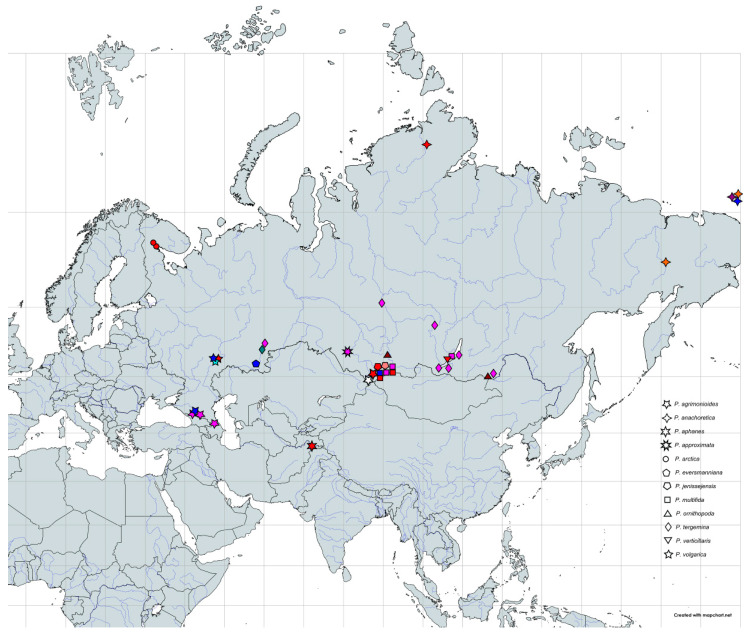
Geographical distribution of studied species and populations of *Potentilla multifida* agg. Colors of symbols follow Figure 1. Closely situated localities of the same species are lumped when represented by the same haplotype lineage. Localities of *P. tergemina* on railroads outside its natural range are not shown.

At the second stage, we reduced the alignment by excluding all the distantly related sequences of the outgroup and analyzed the genealogical relations of the ingroup sequences with the statistical parsimony approach realized in the TCS software. Indels were treated as missing data. The program calculated the 95% parsimony limit of 12 mutational steps and collapsed the sequences into 40 haplotypes united into a single network. Twelve of them were placed into the network as internal haplotypes, connected to two or more neighboring haplotypes. Accordingly, 28 haplotypes were tip haplotypes, connected to a single neighboring haplotype [33]. Further twenty-eight haplotypes were calculated by the program and included into the network as missing hypothetical haplotypes. The network has no loops and is shown in Figure 2. It comprises 12 internal haplotypes designated with capital letters A to L, and 28 tip haplotypes designated with letters and figures. Of course, these haplotype names are conventional and their choice is, to a large extent, driven by the convenience of further network interpretation. The network is unrooted and can be considered as a combination of several variously related haplotype groups, each encompassing internal haplotypes and tip haplotypes derived from them. Their geographical distribution and correspondence to morphological taxa is shown in Figure 3.

The second group represents haplotypes descending from internal haplotype G, differing from haplotype A by one mutational step. They include tip haplotypes G1 to G10 differing from haplotype G by one to four mutational steps, and a lineage comprising internal haplotype H and tip haplotype H1. Internal haplotype G occurs among samples of *P. agrimonioides* from the Caucasus, *P. multifida* from Southern Siberia, and *P. tergemina* from different parts of its area, including the samples occurring as weeds carried along railways in the European part of Russia and Ukraine. As in the previous case, tip haplotypes are rare and found in solitary samples of several species. Haplotypes G1, G5, G6, G8, G9 are found in *P. tergemina*, haplotypes G2, G3, and G4 occur in *P. multifida*, and haplotype G7 is characteristic of one sample of *P. agrimonioides* from the Northern Caucasus. Haplotypes of the H-H1 lineage are exclusively characteristic of *P. anachoretica* from Wrangel Island off the coast of Chukotka in the Arctic Ocean.

The first group of haplotypes comprises tip haplotypes related to internal haplotype A. Most of them differ from internal haplotype A by a single mutational step (A2–A6). Haplotype A1 differs from A by two mutational steps and haplotype A7 differs by six mutational steps. Haplotype A is also ancestral to four lineages encompassing both internal and tip haplotypes, B-B1, C-C1, D-D1, and E-F-F1. The internal haplotype A occurs in populations of *P. volgarica* (Figure 3) but was also found in single samples of *P. agrimonioides* from the Altai Mountains., *P. jenissejensis* from Tyva, and *P. nivea* from the Caucasus. As to tip haplotypes and the clades descendant from the internal haplotype A, the pattern is more complex. Haplotypes A1–A5 were found in single samples of *P. multifida* from Tyva, *P. aphanes* from Southern Tajikistan, *P. volgarica* from Saratov Province, *P. anachoretica* from the Taimyr Peninsula, and *P. nivea* from the Western Caucasus, respectively. Haplotype A6 was uniquely found in multiple samples of *P. arctica* from the shore and islands of the White Sea. Haplotype A7 was found in *P. verticillaris* from the shore of Lake Baikal. The lineages B-B1, C-C1, D-D1, and E-F-F1 appeared to be species specific for *P. anachoretica*, *P. jenisseiensis*, *P. agrimonioides*, and *P. ornithopoda*, respectively.

Internal haplotypes I and J are three and four mutational steps from haplotype G, respectively, and are found in two samples of *P. tergemina*, from a railway in Kiev (Ukraine) and a roadside plant in the Southeastern Urals, respectively.

Internal haplotype K is distanced from haplotype J by four mutational steps. It is central for the third group of haplotypes represented by tip haplotypes K1–K4 and a clade L-L1-L2. Haplotype K is characteristic of *P. eversmanniana* from the Southern Urals. However, it is also found in a sample of *P. multifida* from the Altai Mountains, a sample of *P. anachoretica* from Wrangel Island, and in four samples of *P. volgarica* from a single locality (a chalk hill near Novaya Yablonka) in Saratov Province. Tip haplotypes were found in solitary samples of *P. volgarica* (K1), *P. eversmanniana* (K2), *P. multifida* (K3), and *P. agrimonioides* (K4). The haplotypes of the lineage L-L1-L2 were exclusively found among samples of *P. volgarica*.

To root the network, we reduced the alignment, deleting all identical sequences among accessions of the same species, and analyzed it together with outgroup sequences with the maximal likelihood approach in raxmlGUI to reconstruct the species tree. The resulting tree is shown in Figure 1. Separate species are represented here by one to several accessions corresponding to the haplotypes revealed with TCS. Generally, the tree is congruent with the statistical parsimony-based haplotype network, yet the basal node is weakly supported and unresolved. The basal node forms a polytomy in which accessions from *P. volgarica*, *P. nivea*, *P. jenissejensis*, and *P. agrimonioides* corresponding to A haplotype sequences are positioned on zero (or very close to zero) length branches, thus matching the internal position of the A haplotype in the network (Figure 2). Eight more terminals of the basal polytomy are positioned on non-zero length branches, representing accessions of *P. agrimonioides* (from the Altai Mountains), *P. anachoretica*, *P. aphanes*, *P. arctica*, *P. multifida*, and *P. volgarica*. The basal polytomy also contains three highly (98%) to moderately (81%) supported clades corresponding to the three haplotype lineages derived from the internal haplotype A in the network. These are the clades of *P. anachoretica* (B and B1 haplotypes), *P. jenissejensis* (C and C1), and *P. ornithopoda* (E, F, and F1).

One of the lineages present in the network is not supported by the tree (D-D1). The major clade derived from the basal polytomy is weakly supported and includes all the remaining samples. It also forms a polytomy, and encompasses three samples of *P. agrimonioides*, *P. multifida*, and *P. tergemina* corresponding to the internal haplotype G and positioned on zero length branches. Nine more terminals emerge from the polytomy on mostly short branches representing accessions of *P. multifida*, *P. tergemina*, *P. approximata*, and *P. agrimonioides* (the Caucasus). In addition to these, the polytomy contains two moderately supported clades, one encompassing two accessions of *P. anachoretica* and *P. agrimonioides* (86%) and the other uniting the remaining samples (79%). The latter clade includes a basal grade of two accessions of *P. tergemina* (representing internal haplotypes I and J of the network) and another polytomy uniting samples of *P. eversmanniana*, *P. anachoretica*, *P. volgarica*, *P. multifida*, and *P. agrimonioides* sharing internal haplotype K and all its derivatives.

### 2.2. Nuclear ITS Data Analyses

**Figure 4:**
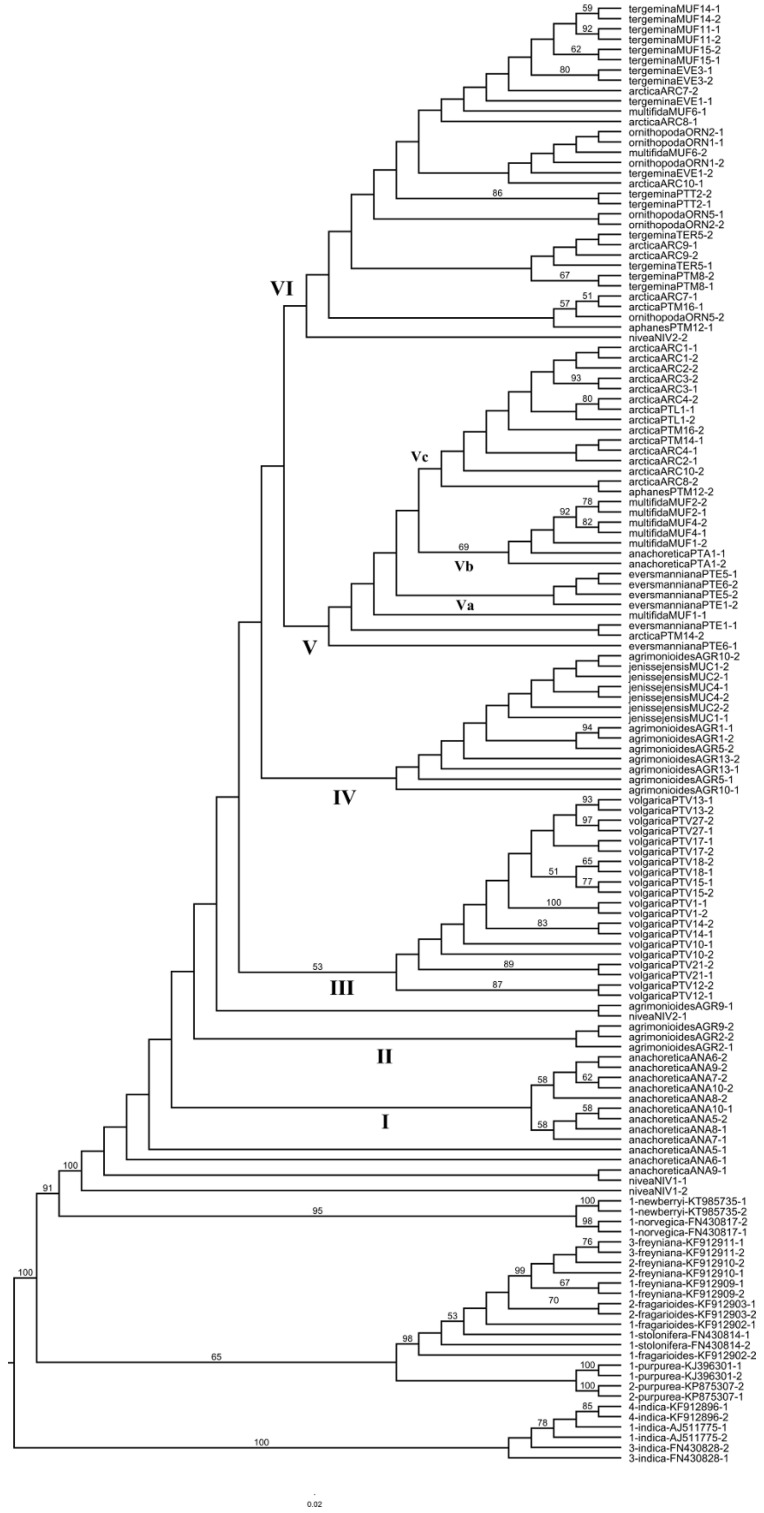
Maximum likelihood tree of *Potentilla multifida* agg. based on ITS data. Terminal names within the ingroup are followed by accession designations as in Table A1 and Figure 1 or Figure 2 indicating reconstructed alleles. Major clades corresponding to species are designated with Roman numerals. Bootstrap support higher than 50% is indicated above branches.

**Figure A1:**
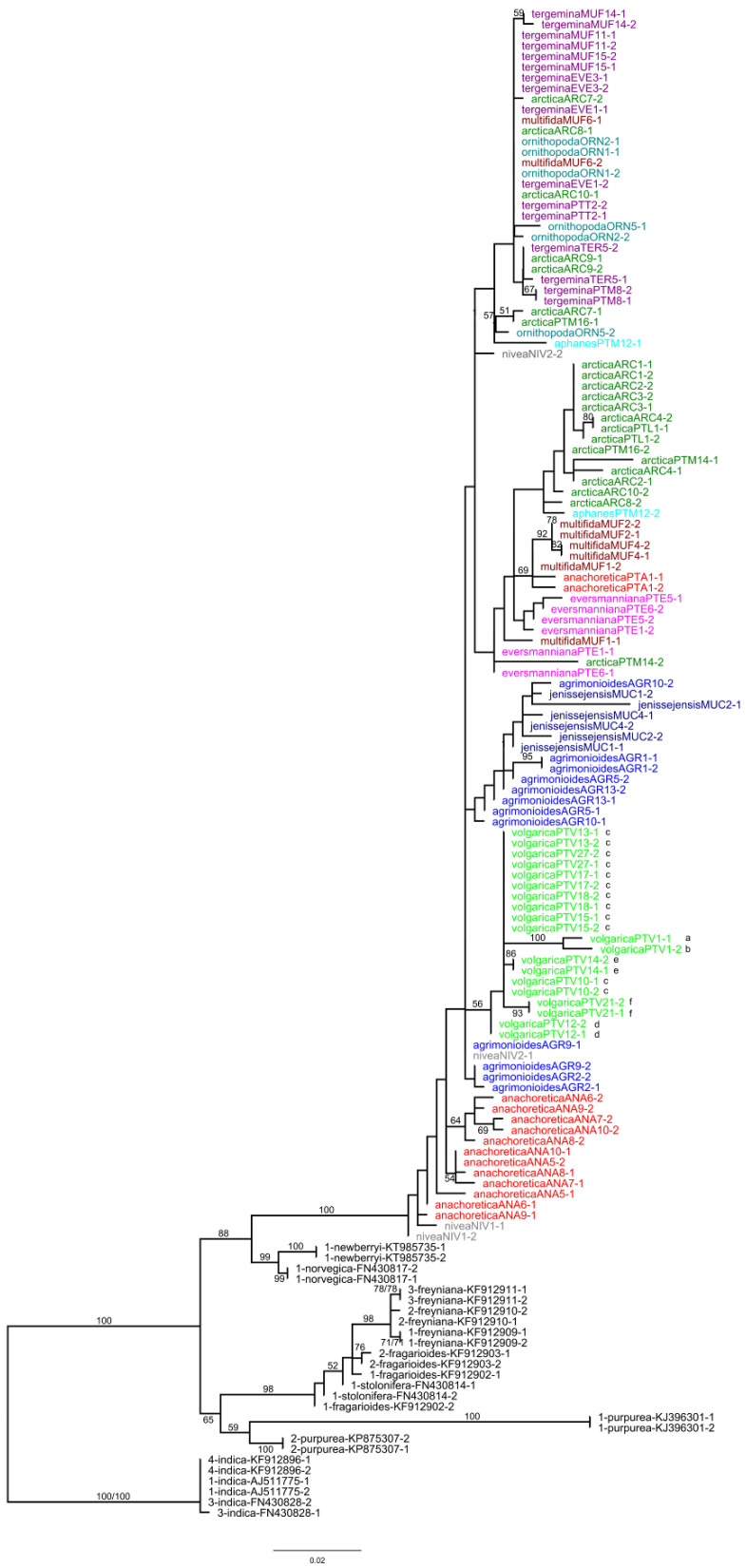
Maximum likelihood tree of *Potentilla multifida* agg. based on ITS data. Terminal names within the ingroup are followed by accession designations as in Table A1 and Figure 1 or Figure 2 indicating reconstructed alleles. Lower-case letters after *P. volgarica* terminals indicate ribotypes of this species. Different species are highlighted with different colors.

We managed to sequence the nuclear ribosomal ITS region from only a subset of samples sequenced for plastid IGS regions (Appendix A, Table A1). Readable parts of the ITS region varied in length from 390 to 521 bp. The alignment length was 529 bp, starting from the motif TTGTCGAA to the motif GAGGCT(T/-)CC, without any major gaps. Thirty-six sequences of the ingroup and five of the outgroup had 1–11 positions with ambiguities due to double peaks in electrophoregrams indicating probable heterozygosity of the samples. Altogether the alignment had 144 polymorphic sites, 71 of which had more than two variants. We did not clone sequences with ambiguities, but reconstructed possible ribotypes using the PHASE algorithm [34,35] as realized in DNAsp. Though plants under study are most probably not diploids (see Introduction), we assumed them to be diploids for the purpose of further analyses. The alignment thus obtained had two sequences per individual, representing reconstructed alleles or ribotypes. We analyzed it using the ML approach in raxmlGUI. The resulting best tree was not fully resolved and many terminal branches were of zero length (Appendix A, Figure A1). The tree was converted to cladogram format for convenience of interpretation (Figure 4). Two different alleles of the same accession are designated with Figure 1 and Figure 2 after a hyphen character in a terminal name. The ingroup forms a monophyletic clade with 100% bootstrap support, with both accessions of *P. nivea* included into the ingroup. Though deeper nodes of the tree are mostly unsupported, it is notable that conspecific accessions here form monophyletic groups with few exceptions. The basal grade includes both alleles of the first accession of *P. nivea* from the Caucasus and the first alleles of three accessions of *P. anachoretica* from Wrangel Island. Their counterparts with all the remaining accessions of this species from Wrangel Island constitute clade I. Clade II comprises two accessions of *P. agrimonioides* from the Caucasus representing both alleles of accession AGR2, and one of the alleles of accession AGR9. The second allele of this accession appears to be in the next (not numbered) clade of the grade together with an allele of the second accession of *P. nivea*. Clade III unites all the accessions of *P. volgarica*. This clade is weakly supported (53%), yet most of its terminal subclades have moderate to high support. Clade IV unites most of the accessions of *P. agrimonioides*, both from the Caucasus and the Altai Mountains, and all the three accessions of *P. jenissejensis*, which form a separate subclade. Clade V unites accessions of several species and includes three major subclades. The subclade Va (unsupported) unites alleles of three accessions of *P. eversmanniana*; the subclade Vb (69% support) unites three accessions of *P. multifida* and one accession of *P. anachoretica* (both alleles); the subclade Vc unites most of the accessions of *P. arctica* from the Kola Peninsula and one of the alleles of *P. aphanes* from Tadjikistan. Clade VI is the most heterogenous and includes accessions of *P. tergemina*, *P. arctica*, *P. multifida*, *P. ornithopoda*, and two alleles of *P. aphanes* and *P. nivea* with their counterparts in different clades.

### 2.3. *Potentilla volgarica* and *P. eversmanniana* Population Structure Analyses

We specifically analyzed populations of *P. volgarica* and *P. eversmanniana* to assess intra- and interpopulation genetic variability. Populations of *P. volgarica* appear to be extremely polymorphic in terms of plastid data and slightly less so in terms of nuclear ITS data, while populations of *P. eversmanniana* show low to zero variability in haplotype compositions (Table A1). The results of AMOVA analyses (Table 1) based on plastid sequences show most of the variability is between in-group populations (i.e., conspecific populations in our case). In the case of populations of *P. volgarica* taken separately, most of the variability is among local populations. The Mantel test demonstrated medium, but significant, correlation between genetic and geographical distances when both species are taken into consideration (r = 0.497, *p* = 0.000). However, correlation between genetic and geographical distances is not significant (r = 0.470, *p* = 0.084) when only populations of *P. volgarica* are considered.

**Figure 5:**
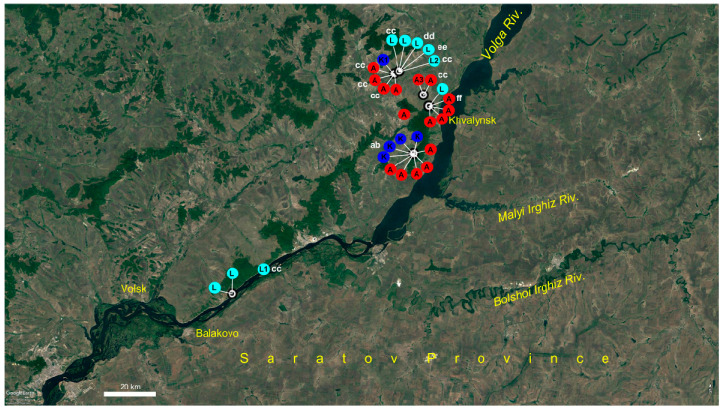
Plastid haplotypes and ITS genotypes geographical distribution in local populations of *Potentilla multifida* agg. Haplotype names follow Figure 2. ITS genotypes are designated with lower-case letters.

Populations of *P. volgarica* are geographically structured at a local scale, haplotypes of the most derived plastid L clade occurring at the extreme south and north of the species area, whereas haplotypes of A and K clades occupy its central part (Figure 5).

## 3. Discussion

Our results suggest *P. multifida* agg. comprises a number of relatively young and incompletely genetically differentiated species widely distributed in Northern Eurasia. Plastid data suggest an incomplete lineage sorting (ILS) characteristic of the group as a whole, including *P. nivea,* traditionally referred to as a different section *Niveae* (Rydb.) A.Nelson. As it is clear from the plastid species tree (Figure 1), *P. nivea* shares the most basal haplotype A with a number of accessions of different species of *P. multifida* agg. The internal basal haplotype A was abundantly sampled from populations of *P. volgarica* only. In addition to these, we managed to reveal the haplotype A only in two accessions of *P. agrimonioides* and *P. jenissejensis* from the Altai Mountains in Altai and Tyva Republics, respectively, and in the above-mentioned accession of *P. nivea* from the Northern Caucasus. At the same time, its derivative tip haplotypes and clades are widely distributed over the whole range of the group. They are however absent from the Caucasus, the Urals, and forested areas of Southern Siberia, mostly occupied by populations bearing haplotypes of derivative G and K haplotype lineages (Figure 1 and Figure 3). The haplotype A clade members appear to prevail in more harsh environments in the north and high mountains in the south. However, generally, no clear geographic pattern can be seen in the distribution of plastid haplotype groups with several instances of distantly-related tip haplotypes occurring in the same population. The absence of a clear distribution pattern supports incomplete lineage sorting and recurrent hybridization. The picture emerging from plastid data is that, much like European *P. crantzii* (Crantz) Beck ex Fritsch [26], the common ancestor of *P. multifida* agg. occupied a wide Eurasian periglacial range during cold periods of the Pleistocene period, and contracted to the modern disjunct distribution of *P. multifida* agg. species with climate warming. Similarly, considerable range expansions during the Last Glacial Maximum, and corresponding range contractions during the last interglacial and Holocene periods were discovered in *Sibbaldia procumbens* s.l. [36] and *Rosa sericea* s.l. complex [37].

Quite surprisingly, nuclear ITS demonstrates a different pattern, with most conspecific accessions, notably *P. anachoretica*, *P. volgarica*, and *P. agrimonioides* together with *P. jenissejensis*, *P. eversmanniana*, *P. multifida*, *P. arctica*, and *P. tergemina,* nesting within monophyletic clades (Figure 4). Several exceptions, where separate alleles of these species fall outside their main clades, appearing mostly in the clade VI representing all the accessions of *P. tergemina*, may indicate hybridization of these species with *P. tergemina* or its direct ancestor in the past. Notably, *P. ornithopoda* inferred alleles are dispersed among subclades of clade VI, while in the plastid tree its accessions form a monophyletic clade of haplotypes E, F, and F1. This may indicate a hybrid origin from unknown parents, probably unsampled in our study.

A special case represents *P. volgarica*. First of all, it is extremely genetically diverse as to plastid haplotypes. Most of the other species considered in this study possess few usually closely related haplotypes, or even are monomorphic, as *P. arctica* (A6), or nearly monomorphic, as *P. eversmanniana* (K, K2). *P. volgarica* is the only species represented by seven different haplotypes, A, A3, K, K1, L, L1, and L2, from two distant haplotype groups (A and K). Though just a subset of samples was sequenced for nrITS, all but one accession appeared to be homozygous, a situation reversed in other species where heterozygotes predominate. This also refers to its probably closest relative, *P. eversmanniana* from the Southwestern Urals. We sampled two local populations of this species (Table 1) and revealed them nearly monomorphic as to plastid haplotypes—all the plants possessed haplotype K, while its close derivative tip haplotype K2 was found in a singe plant. Contrary to that, all three specimens sequenced for ITS appeared to be heterozygotous. Moreover, populations of *P. volgarica* are mostly represented by homozygotes. We have studied seven local populations of *P. volgarica* from all localities of this species known so far (Figure 5). This mosaic pattern of plastid haplotype diversity together with predominant homozygosity of populations by ITS and results of AMOVA and Mantel test is suggestive of facultative apomixis in this species.

The unusual pattern of genetic diversity in *P. volgarica* may be explained by long persistence of the species in its current, extremely small distribution area, which probably behaves as a contemporary refugium. Ecologically, this species is restricted to steppe on hills with chalk outcrops. *Potentilla volgarica* populations, especially those in the central part of the area, deserve protection because they harbor most of the species’ genetic polymorphism. *Potentilla eversmanniana* needs further study with larger sampling. However, the low genetic diversity observed in the present study suggests that it may be more vulnerable to habitat disturbance and climate change, than *P. volgarica*. Moreover, the plastid data suggest that it diverged from *P. volgarica* in the Pleistocene period, when this species probably had a wider distribution area.

## 4. Materials and Methods

### 4.1. Taxon Sampling

The plant material used in the present study was sampled from five local populations in the Saratovskaya Province of Russia (*P. volgarica*) and from two local populations in the Republic of Bashkortostan of Russia (*P. ewersmanniana*) in May–June 2019. Additional samples of both species, as well as *P. arctica* (a two population series in MHA and MW), *P. anachoretica* (a population series in MW and MHA), *P. agrimonioides*, *P. aphanes* Soják, *P. jenissejensis* (three specimens kept in MW under the name *P. multicaulis*), *P. multifida*, *P. ornithopoda*, *P. approximata* Bunge, *P. tergemina* (including several samples outside the natural range of the species collected at railroads in European Russia), and *P. verticillaris* were obtained from herbarium specimens kept at MHA, MW, and SARBG Herbaria. These materials were supplemented by sequences of distantly related species from other sections of *Potentilla* obtained from GenBank and used as an outgroup: *P. freyniana* Bornm. MK209638; *P. hebiichigo* Yonek. & H.Ohashi MK144666; *P. indica* (Andrews) Th.Wolf KY420014, MK134678; *P. purpurascens* (S.Watson) Greene KY419979; *P. purpurea* Hook.f. KY419953, *P. stolonifera* Lehm. ex Ledeb. MK227179; *P. tilingii* (Regel) Greene KY420028. To root nuclear ITS trees we used following sequences acquired from GenBank: *P. fragarioides* L. KF912902, KF912903; *P. freyniana* KF912909, KF912910, KF912911; *P. indica* AY862574, KF912896, AJ511775, FN430828; *P. newberryi* A.Gray KT985735; *P. norvegica* L. FN430817; *P. purpurea* KP875307; *P. stolonifera* FN430814.

We determined the sampled plants using the keys to species of *Potentilla* in “Flora Europae Orientalis” [7], “Monographie der Gattung *Potentilla*” [1], J. Soják’s critical papers [18,19,20], and V. Kurbatsky’s [38] keys to the species of *Potentilla* of Asian Russia based on morphological characters. Two samples of *P. eversmanniana* from MW collected in Sverdlovsk Province (the Central Urals) were redetermined as *P. tergemina*, and three samples of *P. multicaulis* Bunge as *P. jenissejensis*. All the samples used for the analyses are listed in Appendix A, Table A1.

### 4.2. Molecular Methods

Total DNA was extracted from silica-dried leaf tissue and, in some cases, from herbarium samples using the NucleoSpin Plant DNA kit (Macherey Nagel, Germany) according to the manufacturer’s protocol. For the molecular phylogenetic study we used three markers: nuclear ribosomal ITS1 and two plastid intergenic spacers (IGS), *ndhC-trnV* and *psbA-trnH*. For the amplification and subsequent sequencing of ITS region, NNC–18S10 (AGGAGAAGTCGTAACAA) and C26A (GTTTCTTTTCCTCCGCT) primers were used [39]. The plastid *psbA-trnH* IGS was amplified using *trnH* (CGCGCATGGTGGATTCACAATCC) and *psbA* (GTTATGCATGAACGTAATGCTC) primers, and the *ndhC–trnV* IGS was amplified with *ndhC* (ATTAGAAATGYCCARAAAATATCAT) and *trnV(UAC)*x2 (GTCTACGGTTCGARTCCGTA) primers [40,41]. We chose these two regions after a pilot screening of several potentially variable plastid markers [40,41] with a small subset of samples. All PCR products were directly sequenced in both directions with the same primers. Primers used for PCR were synthesized and purified in PAAG by Syntol Ltd. (Moscow, Russia). Double-stranded DNA templates were obtained by polymerase chain reaction (PCR). PCR reaction mixture (20 µL) contained 1.5–2 ng of DNA, 5 pmol of each primer, 4 µL of Ready-to-Use PCR Master mix 5× MasDDTaqMIX-2025, containing a “hot-start” SmarTaq DNA polymerase (Dialat Ltd., Moscow, Russia) and 13 µL of deionized water. PCR reaction was performed on a MJ Research PTC220 DNA Engine Dyad Thermal Cycler (BioRad Laboratories, Foster City, CA, United States) under the following conditions. For ITS: 94 °C for 3 min; 94 °C for 20 s, 58 °C for 30 s, and 72 °C for 40 s (34 cycles); and 72 °C for 3 min. For *ndhC-trnV*: 95 °C for 3 min; 95 °C for 30 s, 57 °C for 40 s, and 60 °C for 1 min 20 s (35 cycles); 57 °C for 40 s, 60 °C for 1 min 20 s (2 cycles). For *psbA-trnH*: 95 °C for 3 min; 95 °C for 30 s, 52, 5 °C for 30 s, and 72 °C for 1 min 30 s (40 cycles); and 72 °C for 7 min. PCR reaction products were separated on 1% agarose gel in 0.5 × TBE buffer containing ethidium bromide and purified by precipitation in 0.125 M/L ammonium acetate solution in 70% ethanol. DNA was sequenced on a 3730 DNA Analyzer (Applied Biosystems, Foster City, CA, United States) at the Genome Research Resource Center, Engelhardt Institute of Molecular Biology. All sequences were deposited in the GenBank database (www.ncbi.nlm.nih.gov); GenBank accession numbers of newly sequenced specimens are compiled in Table A1 (Appendix A).

### 4.3. Sequence Editing and Alignment

For the purposes of analysis, both plastid regions were concatenated. We were not able to sequence the ITS region for all samples (see Table A1); for many samples this region was sequenced incompletely. Sequences were aligned using MAFFT [42,43] with the accurate strategy L-INS-i and modified manually using BioEdit 7.0 [44]. ITS and concatenated chloroplast regions were analyzed separately. Since the plastid alignment had multiple indels, some of which being dubiously aligned, we used the BMGE (Block Mapping and gathering with Entropy) v. 1.1. software [30] with the default –t option to trim the alignment to remove gaps and phylogenetically uninformative and homoplasious positions.

### 4.4. Data Analyses

Phylogenetic reconstruction was performed in RAxML ver. 8.2.10 using raxmlGUI 2.0 beta [45,46,47,48]. Bootstrap values are based on 100 replicates (Fast bootstrap option). The program searched for trees with the maximum likelihood approach under the GTRGAMMA model with parameters calculated by the program. Phylogenetic relationships among the cpDNA haplotypes were reconstructed using statistical parsimony analysis as implemented in TCS v1.2 [49] with alignment gaps treated as missing data. We edited the resulting trees in FigTree v.1.4.3 [50] and finally elaborated all the figures in InkScape v.0.48.2 (https://inkscape.org).

To root the trees we downloaded from GenBank eight complete chloroplast genomes of different species of *Potentilla* and used only two regions from these cp genomes, *ndh*C-*trn*V and *psb*A-*trn*H. Additionally, we sequenced two specimens of *P. nivea* as a possible close outgroup [14].

The programs DNAsp v.6 [51] and Arlequin v. 3.5.2.2. [52] were used to calculate several genetics parameters. For the geographical mapping of *Potentilla* haplotypes we used the Google Earth 7.3.2.5776 software.

## Figures and Tables

**Table 1:** AMOVA results for *Potentilla volgarica* and *P. eversmanniana* based on plastid DNA sequences. Fixation indices significant at *p* < 0.05 are highlighted in bold.

Source of Variation	d.f.	Variance Components	Percentage of Variation	Fixation Indices
*Potentilla volgarica* vs. *P. eversmanniana*				
Among groups	1	3.23474 Va	16.18	FSC = 0.63879
Among populations within groups	7	10.70696 Vb	53.55	**FST = 0.69722**
Within populations	37	6.05440 Vc	30.28	**FCT = 0.16177**
*Potentilla volgarica*				
Among populations	6	13.34836 Va	58.94	**FST = 0.58942**
Within populations	24	9.29815 Vb	41.06	-

## Appendix A

**Table A1:** Species, accessions, specimen vouchers, plastid haplotypes, and GenBank accession numbers of plastid and nuclear ITS sequences used in the study. Underlined ITS accession numbers indicate the presence of ambiguous positions in ITS sequences (putative heterozygotes).

Samples and Accessions	Specimen Voucher	Plastid Haplotype	*ndh*C-*trn*V	*psb*A- *trn*H	ITS
*P. agrimonioides* (AGR1)	[Russia] Karachay-Cherkessia, W spurs of Mt. Elbrus, right slope of the Biytik-Tyubyu Riv. basin, ca. 5 km upstream the Kyukyurtlyu Riv. mouth, meadow-steppe slope, 4.08.2009, A.S. Zernov No. 7269, 43.411297° N, 42.344777° E (MW)	G	MW046910	MW046965	MW042130
*P. agrimonioides* (AGR10)	[Russia] Altai Republic, Kosh-Agachsky distr., Saylyugem ridge, the Chagan-Burgazy Riv. basin, middle part of the left slope of the Bayan-Chagan Riv. valley ca. 5 km upstream its flow into the Karasu Riv. 2690 m alt., 11.08.1999, D.A. Petelin, N.V. Fedotkina No. 99-462 (specimen 2), 49.516667° N, 88.766667° E (MW)	D	MW046906	MW046961	MW042127
*P. agrimonioides* (AGR11)	[Russia] Altai Republic, Kosh-Agachsky distr., Saylyugem ridge, the Chagan-Burgazy Riv. basin, middle part of the left slope of the Bayan-Chagan Riv. valley ca. 5 km upstream its flow into the Karasu Riv. 2690 m alt., 11.08.1999, D.A. Petelin, N.V. Fedotkina No. 99-462 (specimen 3), 49.516667° N, 88.766667° E (MW)	D1	MW046907	MW046962	–
*P. agrimonioides* (AGR12)	[Russia] Altai Republic, Kosh-Agachsky distr., Chikhachev ridge, the Bar-Burgazy Riv. valley, left shingle bank of the river, alt. 2075 m, 05.07.1982, A. Maneev, A. Krasnikov, 49.916667° N, 89.416667° E (MW)	D	MW046908	MW046963	–
*P. agrimonioides* (AGR13)	[Russia] Altai Republic, Kosh-Agachsky distr., Chikhachev ridge, the Tekelyu Riv. valley (left tributary of the Bguzun Riv.), rocks of the left bank, alt. 2300 m, 27.07.1981, A. Maneyev, A. Krasnikov (left sample), 50.083333° N, 89.416667° E (MW)	A	MW046909	MW046964	MW042129
*P. agrimonioides* (AGR2)	[Russia] Karachay-Cherkessia, Karachaevsky distr., vicinity of Verkhnyaya Mara vill., Mount Kyokle-Bashi, S spurs, alt. ca. 1800 m, meadow-steppe slope near summit, 13.07.2009, A.S. Zernov, S.A. Senator No. 7103, 43.775° N, 42.117778° E (MW)	K4	MW046911	MW046966	MW042131
*P. agrimonioides* (AGR3)	[Russia] Stavropol Territory, Elbrus distr., village Uchkulan, upstream of the Kuban Riv., mountain steppe, 17. 07.1967, T.A. Lezhankina, 43.449018° N, 42.088726° E (MW)	G	MW046912	MW046967	MW042132
*P. agrimonioides* (AGR4)	[Russia] North Ossetian ASSR, the Ardon Riv. basin, left slope of Alagir gorge above Nuzal vill., dry stony slopes, alt. 1100 m, 18.05.1977, A.M. Amirkhanov 42.824758° N, 44.019145° E (MW)	G	MW046913	MW046968	MW042133
*P. agrimonioides* (AGR5)	[Russia] North Ossetian ASSR, the Ardon Riv. basin, the Main Dividing Ridge system, W slope of Tseyskiy Ridge above Buron vill., left slope of Alagir gorge, scree SE slopes, alt. 1200 m, 23.05.1977, A.M. Amirkhanov 42.794852° N, 44.02317° E (MW)	G	MW046914	MW046969	MW042134
*P. agrimonioides* (AGR6)	[Russia] North Ossetian ASSR, the Ardon Riv. basin, the Main Dividing Ridge system, W slope of Tseyskiy Ridge above Buron vill., left slope of Alagir gorge, scree SE slopes, alt. 1250 m, 23.05.1977, A.M. Amirkhanov 42.794852° N, 44.002317° E (MW) (left specimen)	G	MW046915	MW046970	MW042135
*P. agrimonioides* (AGR7)	[Russia] North Ossetian ASSR, the Ardon Riv. basin, the Main Dividing Ridge system, W slope of Tseyskiy Ridge above Buron vill., left slope of Alagir gorge, scree SE slopes, alt. 1250 m, 23.05.1977, A.M. Amirkhanov 42.794852° N, 44.002317° E (MW) (right specimen)	G7	MW046916	MW046971	–
*P. agrimonioides* (AGR9)	[Russia] Dagestan Republic, the Mularchay Riv., shoals of the left river bank, alt. 2740–2500 m, 05.08.1989, A.M. Amirkhanov No. 21, 41.28661° N, 47.741299° E (MW)	G	MW046917	MW046972	MW042136
*P. anachoretica* (ANA10)	[Russia] Chukotka AR, Wrangel Island, middle course of the Mamontovaya Riv. (near inflow of Khrustalny and Vesely streams), meadow on Vesely stream valley slope, 12.07.2014, I.N. Pospelov, E.B. Pospelova No. 14-112 (upper right specimen), 71.175511° N, 179.75655° E (MW)	H	MW046918	MW046973	MW042137
*P. anachoretica* (ANA4)	[Russia] Chukotka AR, Wrangel Island, middle course of the Mamontovaya Riv. (near inflow of Khrustalny and Vesely streams), steppe meadow at Vesely stream mouth, 04.07.2014, I.N. Pospelov, E.B. Pospelova No. 14-109 (upper specimen), 71.175511° N, 179.75655 W (MW)	H1	MW046919	MW046974	–
*P. anachoretica* (ANA5)	[Russia] Chukotka AR, Wrangel Island, middle course of the Mamontovaya Riv. (near inflow of Khrustalny and Vesely streams), steppe meadow at Vesely stream mouth, 04.07.2014, I.N. Pospelov, E.B. Pospelova No. 14-109 (lower specimen), 71.175511° N, 179.75655 W (MW)	H	MW046920	MW046975	MW042138
*P. anachoretica* (ANA6)	[Russia] Chukotka AR, Wrangel Island, upstream of the Neizvestnaya Riv., the northern ‘Bobovaya gorka’ area, tundra, 12.07.2014, I.N. Pospelov, E.B. Pospelova No. 14-049 (upper specimen) 71.169347° N, 179.434647 W (MW)	H	MW046921	MW046976	MW042139
*P. anachoretica* (ANA7)	[Russia] Chukotka AR, Wrangel Island, upstream of the Neizvestnaya Riv., the northern ‘Bobovaya gorka’ area, steppe meadow at rocks, 12.07.2014, I.N. Pospelov, E.B. Pospelova No. 14-111 (upper specimen) 71.178525° N, 179.408767W (MW)	–	–	–	MW042140
*P. anachoretica* (ANA8)	[Russia] Chukotka AR, Wrangel Island, upstream of the Neizvestnaya Riv., the northern ‘Bobovaya gorka’ area, thinned meadow, 12.07.2014, I.N. Pospelov, E.B. Pospelova No. 14-049 (left specimen) 71.169931° N, 179.435272 W (MW)	B	MW046922	MW046977	MW042141
*P. anachoretica* (ANA9)	[Russia] Chukotka AR, Wrangel Island, middle course of the Mamontovaya Riv. (near inflow of Khrustalny and Vesely streams), steppe meadow at Vesely stream mouth, 06.07.2014, I.N. Pospelov, E.B. Pospelova No. 14-110 71.176561° N, 179.7578 W (MW)	H	MW046923	MW046978	MW042142
*P. anachoretica* (PTA1)	[Russia] Central Taimyr Peninsula, Byrranga Mountains, middle course of the Bolshaya Bootankaga Riv. near Vetvisty stream inflow, lower part of matted schistose slope, tundra, 16.07.1990, E.B. Pospelova No. 90-472v, 74.31025° N, 98.07938° E (MHA)	A4	MN849358	MN871334	MN872925
*P. anachoretica* (PTA2)	[Russia] Chukotka AR, Wrangel Island, Somnitelnaya Bay, 19.07.1984, B.Yurtsev, 70.979137° N, 179.463733 W (MHA)	K	MN849359	MN871335	–
*P. anachoretica* (PTM11)	[Russia] Magadan Prov., Bilibinsky distr., right bank of the Omolon Riv., 18 km downstream of the Kedon Riv. mouth, steppe slope, 05.07.1976, G.L. Antropova, A.P. Khokhryakov, 65.717199N, 159.079131E (MHA)	B1	MN849377	MN871353	–
*P. aphanes* (PTM12)	Tajikistan, Western Pamir, Ishkashim distr., vicinity of Vigost vill., scrub, alt. ca. 3500 m, 12.07.1983, G.M. Proskuryakova, Z.R. Alferova, 36.7° N, 71.6° E (MHA)	A2	MN849378	MN871354	MN872943
*P. approximata* (PTM6)	[Russia] Novosibirsk Prov., Ordynsky distr., vicinity of Kirza vill., steppe meadow, alt. 200 m, 01.07.1991, I.M. Krasnoborov No. NS56, 54.12° N, 81.40° E (MHA)	G10	MN849385	MN871361	–
*P. arctica* (ARC1)	[Russia] Murmansk Prov., Kandalaksha distr., Tonnye Islands, Oleny archipelago, sea-side meadow on rocks, 22.06.2016, E.V. Kudr, K.A. Savina, 67.11089° N, 32.40611° E (MW)	A6	MW046926	MW046981	MW042145
*P. arctica* (ARC10)	[Russia] Murmansk Prov., Terskiy distr., White Sea, Kandalaksha Gulf, Porya Bay, Perunok island, southern rocky cape, rocky meadow, 10.07.2011, M.N. Kozhin No. M-1775, 66.77747° N, 33.62591° E (MW)	A6	MW046924	MW046979	MW042143
*P. arctica* (ARC11)	[Russia] Murmansk Prov., White Sea, Porya Bay, Karbonatnaya Luda island, 15.07.2008, M.N. Kozhin No. M-0623, 66.73993° N, 33.63885° E (MW)	A6	MW046925	MW046980	–
*P. arctica* (ARC2)	[Russia] Murmansk Prov., Kandalaksha distr., Kandalaksha Gulf of the White Sea, Luven’gsky archipelago, Golomyanny Vlasov island, cracked sea-side rocks, 16.08.2017, E.I. Vuzman, 67.07184° N, 32.72459° E (MW)	A6	MW046927	MW046982	MW042146
*P. arctica* (ARC3)	[Russia] Murmansk Prov., Kandalaksha distr., Tonnye Islands, Oleny archipelago, sea-side meadow on rocks, 22.06.2016, T.S. Taskina, 67.11089° N, 32.40611° E (MW)	A6	MW046928	MW046983	MW042147
*P. arctica* (ARC4)	[Russia] Karelia, Loukhsky distr., Vysoky Island, S shore, on rocks, 4.07.2003, L.A. Abramova, P.A. Volkova, K.A. Astafjev, 66.574624° N, 32.90934° E (MW)	–	–	–	MW042148
*P. arctica* (ARC6)	[Russia] Murmansk Prov., Terskiy distr., White Sea, Kandalaksha Gulf, Porya Bay, Chayachya Luda Island, SW part, rock crevices, 4.08.2011, M.N. Kozhin No. M-1858, 66.78037° N, 33.57952° E (MW)	A6	MW046929	MW046984	–
*P. arctica* (ARC7)	[Russia] Murmansk Prov., Terskiy distr., White Sea, Kandalaksha Gulf, Porya Bay, Krachny Baklysh Island, seashore rocks, 14.07.2011, M.N. Kozhin No. M-2075, 66.78828° N, 33.63157° E (MW)	A6	MW046930	MW046985	MW042149
*P. arctica* (ARC8)	[Russia] Murmansk Prov., Terskiy distr., White Sea, Kandalaksha Gulf, Porya Bay, Medvezhy Island, SE cape, rocks, 21.07.2008, M.N. Kozhin No. M-0601, 66,7° N, 33,6° E (MW)	A6	MW046931	MW046986	MW042150
*P. arctica* (ARC9)	[Russia] Murmansk Prov., Terskiy distr., White Sea, Kandalaksha Gulf, Porya Bay, Chayachya Luda Island, SW part, rock crevices, 4.08.2011, M.N. Kozhin No. M-1858 (2^nd^ sheet), 66.78037° N, 33.57952° E (MW)	A6	MW046932	MW046987	MW042151
*P. arctica* (PTL1)	[Russia] Murmansk Prov., Kandalaksha distr., 2.5 km NE of Kovda vill., Ovechy Island, seashore, in rock crevices, 30.07.2004, N.M. Reshetnikova, 66.7° N, 39.2° E (MHA)	A6	MN849376	MN871352	MN872940
*P. arctica* (PTM14)	[Russia] Murmansk Prov., Kandalaksha distr., vicinity of Kovda vill., Sedlovaty Island, seashore rocks, 27.07.2019, E.G. Petrash, 66.690052° N, 33.060953° E (1st sheet) (MHA)	A6	MN849379	MN871355	MN872944
*P. arctica* (PTM15)	[Russia] Murmansk Prov., Kandalaksha distr., vicinity of Kovda vill., Sedlovaty Island, seashore rocks, 27.07.2019, E.G. Petrash, 66.690052° N, 33.060953° E (2nd sheet) (MHA)	A6	MN849380	MN871356	–
*P. arctica* (PTM16)	[Russia] Murmansk Prov., Kandalaksha distr., vicinity of Kovda vill., Sedlovaty Island, seashore rocks, 27.07.2019, E.G. Petrash, 66.690052° N, 33.060953° E (3rd sheet) (MHA)	A6	MN849381	MN871357	MN872946
*P. arctica* (PTM17)	[Russia] Murmansk Prov., Kandalaksha distr., vicinity of Kovda vill., Sedlovaty Island, seashore rocks, 27.07.2019, E.G. Petrash, 66.690052° N, 33.060953° E (4th sheet) (MHA)	A6	MN849382	MN871358	–
*P. eversmanniana* (PTE1)	[Russia] Rep. of Bashkortostan, Zianchurinskii distr., Bashkirskaya Chumaza vill., ca. 500 m downstream the Bolshaya Samaza Rivulet, flat place on a hill above the village, overgrazed feather-grass steppe, 26.05.2019, I.A. Schanzer, A.V. Fedorova No. SH260519-13, 51.943102° N, 56.771153° E (MHA)	K	MN849367	MN871343	MN872926
*P. eversmanniana* (PTE10)	[Russia] Rep. of Bashkortostan, Zianchurinskii distr., ca. 2 km S of Bashkirskaya Chumaza vill., a small valley between hills, stony slopes at a hill summit, 27.05.2019, I.A. Schanzer, A.V. Fedorova No. SH270519-02, 51. 922852° N, 56. 767177° E (MHA)	K	MN849360	MN871336	–
*P. eversmanniana* (PTE11)	[Russia] Rep. of Bashkortostan, Zianchurinskii distr., ca. 2 km S of Bashkirskaya Chumaza vill., a small valley between hills, stony slopes at a hill summit, 27.05.2019, I.A. Schanzer, A.V. Fedorova No. SH270519-03, 51. 922852° N, 56. 767177° E (MHA)	K	MN849361	MN871337	–
*P. eversmanniana* (PTE12)	[Russia] Rep. of Bashkortostan, Zianchurinskii distr., ca. 2 km S of Bashkirskaya Chumaza vill., a small valley between hills, stony slopes at a hill summit, 27.05.2019, I.A. Schanzer, A.V. Fedorova No. SH270519-04, 51. 922852° N, 56. 767177° E (MHA)	K2	MN849362	MN871338	–
*P. eversmanniana* (PTE13)	[Russia] Rep. of Bashkortostan, Zianchurinskii distr., ca. 2 km S of Bashkirskaya Chumaza vill., a small valley between hills, stony slopes at a hill summit, 27.05.2019, I.A. Schanzer, A.V. Fedorova No. SH270519-05, 51. 922852° N, 56. 767177° E (MHA)	K	MN849363	MN871339	–
*P. eversmanniana* (PTE14)	[Russia] Rep. of Bashkortostan, Zianchurinskii distr., ca. 2 km S of Bashkirskaya Chumaza vill., a small valley between hills, stony slopes at a hill summit, 27.05.2019, I.A. Schanzer, A.V. Fedorova No. SH270519-06, 51. 922852° N, 56. 767177° E (MHA)	K	MN849364	MN871340	–
*P. eversmanniana* (PTE15)	[Russia] Rep. of Bashkortostan, Zianchurinskii distr., ca. 2 km S of Bashkirskaya Chumaza vill., a small valley between hills, stony slopes at a hill summit, 27.05.2019, I.A. Schanzer, A.V. Fedorova No. SH270519-07, 51. 922852° N, 56. 767177° E (MHA)	K	MN849365	MN871341	–
*P. eversmanniana* (PTE2)	[Russia] Rep. of Bashkortostan, Zianchurinskii distr., Bashkirskaya Chumaza vill., ca. 500 m downstream the Bolshaya Samaza Rivulet, flat place on a hill above the village, overgrazed feather-grass steppe, 26.05.2019, I.A. Schanzer, A.V. Fedorova No. SH260519-14, 51.943102° N, 56.771153° E (MHA)	K	MN849368	MN871344	–
*P. eversmanniana* (PTE3)	[Russia] Rep. of Bashkortostan, Zianchurinskii distr., Bashkirskaya Chumaza vill., ca. 500 m downstream the Bolshaya Samaza Rivulet, flat place on a hill above the village, overgrazed feather-grass steppe, 26.05.2019, I.A. Schanzer, A.V. Fedorova No. SH260519-15, 51.943102° N, 56.771153° E (MHA)	K	MN849369	MN871345	–
*P. eversmanniana* (PTE4)	[Russia] Rep. of Bashkortostan, Zianchurinskii distr., Bashkirskaya Chumaza vill., ca. 500 m downstream the Bolshaya Samaza Rivulet, flat place on a hill above the village, overgrazed feather-grass steppe, 26.05.2019, I.A. Schanzer, A.V. Fedorova No. SH260519-16, 51.943102° N, 56.771153° E (MHA)	K	MN849370	MN871346	–
*P. eversmanniana* (PTE5)	[Russia] Rep. of Bashkortostan, Zianchurinskii distr., Bashkirskaya Chumaza vill., ca. 500 m downstream the Bolshaya Samaza Rivulet, flat place on a hill above the village, overgrazed feather-grass steppe, 26.05.2019, I.A. Schanzer, A.V. Fedorova No. SH260519-17, 51.943102° N, 56.771153° E (MHA)	K	MN849371	MN871347	MN872930
*P. eversmanniana* (PTE6)	[Russia] Rep. of Bashkortostan, Zianchurinskii distr., Bashkirskaya Chumaza vill., ca. 500 m downstream the Bolshaya Samaza Rivulet, flat place on a hill above the village, overgrazed feather-grass steppe, 26.05.2019, I.A. Schanzer, A.V. Fedorova No. SH260519-18, 51.943102° N, 56.771153° E (MHA)	K	MN849372	MN871348	MN872931
* P. eversmanniana * (PTE7)	[Russia] Rep. of Bashkortostan, Zianchurinskii distr., Bashkirskaya Chumaza vill., ca. 500 m downstream the Bolshaya Samaza Rivulet, flat place on a hill above the village, overgrazed feather-grass steppe, 26.05.2019, I.A. Schanzer, A.V. Fedorova No. SH260519-26, 51.943102° N, 56.771153° E (MHA)	K	MN849373	MN871349	–
* P. eversmanniana * (PTE8)	[Russia] Rep. of Bashkortostan, Zianchurinskii distr., Bashkirskaya Chumaza vill., ca. 500 m downstream the Bolshaya Samaza Rivulet, flat place on a hill above the village, overgrazed feather-grass steppe, 26.05.2019, I.A. Schanzer, A.V. Fedorova No. SH260519-27, 51.943102° N, 56.771153° E (MHA)	K	MN849374	MN871350	–
*P. eversmanniana* (PTE9)	[Russia] Rep. of Bashkortostan, Zianchurinskii distr., ca. 2 km S of Bashkirskaya Chumaza vill., a small valley between hills, stony slopes at a hill summit, 27.05.2019, I.A. Schanzer, A.V. Fedorova No. SH270519-01, 51. 922852° N, 56. 767177° E (MHA)	K	MN849375	MN871351	–
*P. jenissejensis* (MUC1)	[Russia] Altai, Kosh-Agachsky distr., Chikhachev ridge, the Bar-Burgazy Riv. valley, thinned grassy slope in the flood plain, alt. 2100 m, 5.07.1982, A. Maneyev, A. Krasnikov, 49.916667° N, 89.416667° E (1st sheet) (MW)	C	MW046936	MW046991	MW042156
*P. jenissejensis* (MUC2)	[Russia] Tuvinskaya ASSR, Mongun-Taiginsky distr., Chikhachev ridge, the Ustu-Gimateh Riv. valley, talus, 11.08.1980, A. Krasnikov, A. Maneyev, N. Sidorenko (right specimen), 50.453164° N, 89.991742° E (MW)	A	MW046937	MW046992	MW042157
*P. jenissejensis* (MUC4)	[Russia] Altai, Kosh-Agachsky distr., Chikhachev ridge, the Bar-Burgazy Riv. valley, thinned grassy slope in the flood plain, alt. 2100 m, 5.07.1982, A. Maneyev, A. Krasnikov, 49.916667° N, 89.416667° E (2nd sheet) (MW)	C1	MW046939	MW046994	MW042158
*P. multifida* (MUF1)	[Russia] Tuvinskaya ASSR, Mongun-Taiginsky distr., Chikhachev ridge, the Alty-Gimateh Riv. valley 2 km upstream the mouth, meadow, 04.08.1980, A. Krasnikov, A. Maneyev, V. Shein, 50.453164° N, 89.991742° E (MW)	A1	MW046944	MW046999	MW042162
*P. multifida* (MUF13)	[Russia] Irkutsk Prov., [Lake Baikal], Olkhon Island, 2 km° E of Kharantsy vill., shore of the ‘Minor Sea’, 09.07.1979, students, 53.238731° N, 107.459352° E (MW)	G4	MW046941	MW046996	–
*P. multifida* (MUF2)	[Russia] Krasnoyarsky Territory, W Sayan Mountains, Sayano-Shushensky Nature Reserve, left bank of the B. Ura Riv., meadow steppe, alt. 1200 m, 12.08.1988, V. Kuvaev No. 1214-18 (right specimen), 51.990188° N, 91.842219° E (MW)	G	MW046945	MW047000	MW042163
*P. multifida* (MUF4)	[Russia] Tuvinskaya ASSR, Mongun-Taiginsky distr., Chikhachev ridge, the Alty-Gimateh Riv. valley 2 km upstream the mouth, pebble bank, 01.08.1980, A. Krasnikov, A. Maneyev, 50.453164° N, 89.991742° E (MW)	G	MW046946	MW047001	MW042164
*P. multifida* (MUF5)	[Russia] Altai, Kosh-Agachsky distr., Chikhachev ridge, the Baylugem Riv. valley, alt. 2270 m, 21.06.1982, A. Maneyev, A. Krasnikov (upper specimen) 50.25° N, 89.33° E (MW)	G	MW046947	MW047002	–
*P. multifida* (MUF6)	[Russia] Altai, Kosh-Agachsky distr., Chikhachev ridge, the Baylugem Riv. valley, alt. 2270 m, 21.06.1982, A. Maneyev, A. Krasnikov (middle specimen) 50.25° N, 89.33° E (MW)	G3	MW046948	MW047003	MW042165
*P. multifida* (MUF7)	[Russia] Altai, Kosh-Agachsky distr., Chikhachev ridge, the Baylugem Riv. valley, alt. 2270 m, 21.06.1982, A. Maneyev, A. Krasnikov (lower specimen) 50.25° N, 89.33° E (MW)	G2	MW046949	MW047004	–
*P. multifida* (PTM3)	[Russia] Altai, Kosh-Agachsky distr., the Tarhata Riv. valley, meadow on river bank, alt. 2150 m, 08.07.1982, M. Lomonosova, A. Vanyaev, 49.45° N, 88.30° E (MHA)	K	MN849383	MN871359	–
*P. multifida* (PTM4)	[Russia] Altai Mountains, vicinity of Kosh-Agach, a quarry in Leymus-steppe, alt. 1770 m, 20.07.1982, I. Krasnoborov, A. Krasnikov No. 1309, 50.00° N, 88.40° E (MHA)	K3	MN849384	MN871360	–
*P. nivea* (NIV1)	[Russia] Karachay-Cherkessia, Teberdinsky State Reserve, N spurs of Malaya Khatipara Mt., stony slope, 12.08.2006, A.S. Zernov No. 5524, 43.438549° N, 41.681227° E (MW)	A	MW046952	MW047007	MW042167
*P. nivea* (NIV2)	[Russia] Karachay-Cherkessia, Karachaevsky distr., S slope of Sadyrla range, alt. ca. 2900, talus, 9.08.2011, A.S. Zernov, A.N. Filin No. 7619, 43.434741° N, 42.266179° E (MW)	A5	MW046951	MW047006	MW042166
*P. ornithopoda* (ORN1)	[Russia] Khakassia Rep., Altaiskyi distr., less than 1 km S of Izykhskiye Kopi vill., alt. ca. 322 m., shallow gully, on wet salty loamy soil, I. Schanzer, N. Stepanova No. 11IK75, 53.539° N, 91.28675° E (MHA)	F	MW046953	MW047008	MW042168
*P. ornithopoda* (ORN2)	[Russia] Khakassia Rep., Altaiskyi distr., less than 1 km S of Izykhskiye Kopi vill., alt. ca. 322 m., shallow gully, on wet salty loamy soil, I. Schanzer, N. Stepanova No. 11IK83, 53.539° N, 91.28675° E (MHA)	E	MW046954	MW047009	MW042169
*P. ornithopoda* (ORN5)	[Russia] Chitinskaya Prov., Ononsky distr., near lake Zun-Torey, 24.07.1977, T. Sofeykova et al., 50,023955° N, 115,907498° E (MHA)	F1	MW046956	MW047011	MW042170
*P. tergemina *(EWE1)	[Russia] Sverdlovskaya Prov., Nevyansk distr., vicinity of Murzinka railway station, 4 km south of lake Tavatuy, 10.06.1991, N. Shvedchikova, 57.055421° N, 60.174235° E (1st sheet) (MW)	G	MW046933	MW046988	MW042153
*P. tergemina *(EWE3)	[Russia] Sverdlovskaya Prov., Nevyansk distr., vicinity of Murzinka railway station, 4 km south of lake Tavatuy, 10.06.1991, N. Shvedchikova, 57.055421° N, 60.174235° E (3rd sheet) (MW)	G	MW046934	MW046989	MW042154
*P. tergemina *(EWE4)	[Russia] Southern Urals, between sources of the Ui and Miass Rivers, field road ca. 1.5 km NW of Starobayramovo vill., Uchalinsky distr. of Bashkortostan, 16.07.1993, M.S. Knyazev 54,740913° N, 59,742309° E (MW)	J	MW046935	MW046990	MW042155
*P. tergemina* (MUF11)	[Russia] Trans-Baikal Territory, Aleksandrovo-Zavodsky distr., near Mankovo vill., the Verkhniy Gazimur Riv. flood plain, steppe meadow, alt. 885 m, 22.07.2011, N.S. Gamova, S.V. Dudov No. 11_0010, 50.8184° N, 117.6888° E (MW)	G	MW046940	MW046995	MW042159
*P. tergemina* (MUF14)	[Russia] Irkutsk Prov., Ust-Ilimsk distr., vicinity of Ust-Ilimsk, right bank of the Ust-Ilimsk reservoir, 4.5 km° E of the railway station, groundwork edge, 09.08.2007, A. Seregin, A. Khokhlov No. S-303, 57.916667° N, 102.852778° E (MW)	G5	MW046942	MW046997	MW042160
*P. tergemina* (MUF15)	[Russia] Irkutsk Prov., Slyudyansky distr., 23 km to° E from Baikalsk, S shore of Lake Baikal, at the Pankovka Riv. mouth, concrete plates at the railway basement, alt. 240 m, 29.07.2007, A. Seregin No. S-15 51.461111° N, 104.491667° E (MW)	G6	MW046943	MW046998	MW042161
*P. tergemina* (MUF9)	[Russia] Republic of Buryatia, Barguzinsky distr., near Ust-Barguzin vill., right slope of the Barguzin Riv. valley, abandoned field, 24.05.2011, S.V. Dudov No. 2011-Bur-022, 53.4431° N, 109.0754° E (MW)	G	MW046950	MW047005	–
*P. tergemina* (PTM8)	[Russia, Krasnoyarsky Territory, Turukhansky distr.] Left bank of the Yenisei Riv., Zotino vill., meadow, 15.07.2001, V. Kuvaev, M. Skrebtsova, A. Sidorov No. 2887-13, 60.901355° N 89.680666° E (MHA)	G	MN849386	MN871362	MN872942
*P. tergemina* (PTM9)	[Russia] Udmurtskaya ASSR, Glazov-Tuktym section of the railroad, 19.06.1983, A.N. Puzyrev, 58.097 ° N, 52.863° E (MHA)	G	MN849387	MN871363	–
*P. tergemina* (PTT1)	[Russia] Buryatia, vicinity of Gusinoozersk, steppe, 04.08.1993, N. Shevyriova, T. Konovalova, 51.17 ° N, 106.32° E (MHA)	G1	MN849388	MN871364	–
*P. tergemina* (PTT2)	[Ukraine] Kiev, Kiev-Tovarnaya railway station, on gravel along tracks, 28.04.1990, S.L. Mosyakin, 50.428762° N, 30.506669° E (MHA)	I	MN849389	MN871365	MN872948
*P. tergemina* (TER1)	[Russia] Tverskaya Prov., Bologovsky distr., vicinity of Kuzhenkino railway station, siding tracks, 10.07.2004, A. Notov, N. Markelova, 57.727525° N, 33.975539° E (MW)	G8	MW046957	MW047012	–
*P. tergemina* (TER3)	[Russia] Moscow, near Kanatchikovo railway station of the Moscow Circle Railway, on groundwork, 24.06.1997. S.R. Mayorov, D.D. Sokolov, 55.70139° N, 37.591641° E (MW)	G	MW046958	MW047013	–
*P. tergemina* (TER5)	[Russia] Moscow, sandy areas of the embankment of the Leningrad railway between stations NATI and Mosselmash, 14.06.1980, M.S. Ignatov, 55.855475° N, 37.543115° E (MW)	G9	MW046959	MW047014	MW042171
*P. verticillaris* (VER1)	[Russia] Irkutskaya Prov., W coast of Lake Baikal, S end of Olkhon Island, steppe, 24.08.1993, N. Shevyreva, T. Konovalova, 53,05° N, 106,966667° E (MHA)	A7	MW046960	MW047015	–
*P. volgarica* (PTE16)	[Russia] Saratovskaya Prov., Khvalynsky distr., Sosnovaya Maza, 14.07.1993, A.K. Skvortsov, 52.505911° N, 47.903532° E (MHA)	A	MN849366	MN871342	–
*P. volgarica* (PTV1)	[Russia] Saratovskaya Prov., Khvalynsky distr., near Novaya Yablonka vill., chalk hill slope over the Syzran-Saratov highway, I. Schanzer et al. No. SH200519-18, 20.05.2019, 52,372381° N, 47,967634° E (MHA)	K	MN849400	MN871376	MN872949
*P. volgarica* (PTV10)	[Russia] Saratovskaya Prov., Khvalynsky distr., Piche-Panda Piche-Panda upland, at the border of feather-grass lined steppe at the hill summit and an upper gentle part of the S slope, I. Schanzer et al. No. SH210519-17, 21.05.2019, 52.654234° N, 47.861662° E (MHA)	L2	MN849390	MN871366	MN872965
*P. volgarica* (PTV11)	[Russia] Saratovskaya Prov., Khvalynsky distr., Piche-Panda Piche-Panda upland, at the border of feather-grass lined steppe at the hill summit and an upper gentle part of the S slope, I. Schanzer et al. No. SH210519-18, 21.05.2019, 52.654234° N, 47.861662° E (MHA)	L	MN849391	MN871367	–
*P. volgarica* (PTV12)	[Russia] Saratovskaya Prov., Khvalynsky distr., Piche-Panda Piche-Panda upland, at the border of feather-grass lined steppe at the hill summit and an upper gentle part of the S slope, I. Schanzer et al. No. SH210519-19, 21.05.2019, 52.654234° N, 47.861662° E (MHA)	L	MN849392	MN871368	MN872951
*P. volgarica* (PTV13)	[Russia] Saratovskaya Prov., Khvalynsky distr., Piche-Panda Piche-Panda upland, at the border of feather-grass lined steppe at the hill summit and an upper gentle part of the S slope, I. Schanzer et al. No. SH210519-20, 21.05.2019, 52.654234° N, 47.861662° E (MHA)	L	MN849393	MN871369	MN872952
*P. volgarica* (PTV14)	[Russia] Saratovskaya Prov., Khvalynsky distr., Piche-Panda Piche-Panda upland, at the border of feather-grass lined steppe at the hill summit and an upper gentle part of the S slope, I. Schanzer et al. No. SH210519-21, 21.05.2019, 52.654234° N, 47.861662° E (MHA)	L	MN849394	MN871370	MN872953
*P. volgarica* (PTV15)	[Russia] Saratovskaya Prov., Khvalynsky distr., Piche-Panda upland, hills near Yeriomkino vill., low deforestated chalky hill, 21.05.2019, I. Schanzer, et al. No. SH210519-28, 52.645109° N, 47.834526° E	K1	MN849395	MN871371	MN872954
*P. volgarica* (PTV16)	[Russia] Saratovskaya Prov., Khvalynsky distr., Piche-Panda upland, hills near Yeriomkino vill., low deforestated chalky hill, 21.05.2019, I. Schanzer, et al. No. SH210519-29, 52.645109° N, 47.834526° E	A	MN849396	MN871372	–
*P. volgarica* (PTV17)	[Russia] Saratovskaya Prov., Khvalynsky distr., Piche-Panda upland, hills near Yeriomkino vill., low deforestated chalky hill, 21.05.2019, I. Schanzer, et al. No. SH210519-29, 52.645109° N, 47.834526° E	A	MN849397	MN871373	MN872956
*P. volgarica* (PTV18)	[Russia] Saratovskaya Prov., Khvalynsky distr., Piche-Panda upland, hills near Yeriomkino vill., low deforestated chalky hill, 21.05.2019, I. Schanzer, et al. No. SH210519-30, 52.645109° N, 47.834526° E	A	MN849398	MN871374	MN872957
*P. volgarica* (PTV19)	[Russia] Saratovskaya Prov., Khvalynsky distr., Piche-Panda upland, hills near Yeriomkino vill., low deforestated chalky hill, 21.05.2019, I. Schanzer, et al. No. SH210519-31, 52.645109° N, 47.834526° E	A	MN849399	MN871375	–
*P. volgarica* (PTV2)	[Russia] Saratovskaya Prov., Khvalynsky distr., near Novaya Yablonka vill., chalk hill slope over the Syzran-Saratov highway, I. Schanzer et al. No. SH200519-19, 20.05.2019, 52.372381° N, 47.967634° E (MHA)	A	MN849411	MN871387	–
*P. volgarica* (PTV20)	[Russia] Saratovskaya Prov., Khvalynsky distr., 4.5 km NW of Khvalynsk, chalk hills near a quarry N of the Balakovo-Syzran highway. 22.05.2019 I. Schanzer et al. No. SH-220519-18, 52.538878° N, 48.040852° E (MHA)	A	MN849401	MN871377	–
*P. volgarica* (PTV21)	[Russia] Saratovskaya Prov., Khvalynsky distr., 4.5 km NW of Khvalynsk, chalk hills near a quarry N of the Balakovo-Syzran highway. 22.05.2019 I. Schanzer et al. No. SH-220519-19, 52.538878° N, 48.040852° E (MHA)	A	MN849402	MN871378	MN872959
*P. volgarica* (PTV22)	[Russia] Saratovskaya Prov., Khvalynsky distr., 4.5 km NW of Khvalynsk, chalk hills near a quarry N of the Balakovo-Syzran highway. 22.05.2019 I. Schanzer et al. No. SH-220519-20, 52.538878° N, 48.040852° E (MHA)	L	MN849403	MN871379	–
*P. volgarica* (PTV23)	[Russia] Saratovskaya Prov., Khvalynsky distr., 4.5 km NW of Khvalynsk, chalk hills near a quarry N of the Balakovo-Syzran highway. 22.05.2019 I. Schanzer et al. No. SH-220519-21, 52.538878° N, 48.040852° E (MHA)	A	MN849404	MN871380	–
*P. volgarica* (PTV24)	[Russia] Saratovskaya Prov., Khvalynsky distr., 4.5 km NW of Khvalynsk, chalk hills near a quarry N of the Balakovo-Syzran highway. 22.05.2019 I. Schanzer et al. No. SH-220519-22, 52.538878° N, 48.040852° E (MHA)	A	MN849405	MN871381	–
*P. volgarica* (PTV25)	[Russia] Saratovskaya Prov., Khvalynsky distr., 4.5 km NW of Khvalynsk, chalk hills near a quarry N of the Balakovo-Syzran highway. 22.05.2019 I. Schanzer et al. No. SH-220519-23, 52.538878° N, 48.040852° E (MHA)	A	MN849406	MN871382	–
*P. volgarica* (PTV26)	[Russia] Saratovskaya Prov., Khvalynsky distr., chalk hills near a road to Popovka, 16.05.1983, L.H., 52.576915° N, 48.006993° E (1st specimen) (SARBG 1498)	A3	MN849407	MN871383	–
*P. volgarica* (PTV27)	[Russia] Saratovskaya Prov., Khvalynsky distr., chalk hills near a road to Popovka, 16.05.1983, L.H., 52.576915° N, 48.006993° E (2nd specimen) (SARBG 1498)	A	MN849408	MN871384	MN872962
*P. volgarica* (PTV28)	[Russia] Saratovskaya Prov., Khvalynsky distr., 3–4 km N of Voskresensk, chalk outcrops of the bank of the Volga Riv., 25.06.1988, E.A. Kireev, 51.852827° N, 46.991139° E (1st specimen) (SARBG 1499)	L	MN849409	MN871385	–
*P. volgarica* (PTV29)	[Russia] Saratovskaya Prov., Khvalynsky distr., 3–4 km N of Voskresensk, chalk outcrops of the bank of the Volga Riv., 25.06.1988, E.A. Kireev, 51.852827° N, 46.991139° E (2nd specimen) (SARBG 1499)	L	MN849410	MN871386	–
*P. volgarica* (PTV3)	[Russia] Saratovskaya Prov., Khvalynsky distr., near Novaya Yablonka vill., chalk hill slope over the Syzran-Saratov highway, I. Schanzer et al. No. SH200519-20, 20.05.2019, 52.372381° N, 47.967634° E (MHA)	K	MN849413	MN871389	–
*P. volgarica* (PTV30)	[Russia] Saratovskaya Prov., Volsky distr., 5 km S of Rybnoye vill., 27.06.1988, E.A. Kireev, 51.944471° N, 47.158729° E (SARBG 1500)	L1	MN849412	MN871388	MN872964
*P. volgarica* (PTV4)	[Russia] Saratovskaya Prov., Khvalynsky distr., near Novaya Yablonka vill., chalk hill slope over the Syzran-Saratov highway, I. Schanzer et al. No. SH200519-21, 20.05.2019, 52.372381° N, 47.967634° E (MHA)	K	MN849414	MN871390	–
*P. volgarica* (PTV5)	[Russia] Saratovskaya Prov., Khvalynsky distr., near Novaya Yablonka vill., chalk hill slope over the Syzran-Saratov highway, I. Schanzer et al. No. SH200519-22, 20.05.2019, 52.372381° N, 47.967634° E (MHA)	K	MN849415	MN871391	–
*P. volgarica* (PTV6)	[Russia] Saratovskaya Prov., Khvalynsky distr., near Novaya Yablonka vill., chalk hill slope over the Syzran-Saratov highway, I. Schanzer et al. No. SH200519-23, 20.05.2019, 52.372381° N, 47.967634° E (MHA)	A	MN849416	MN871392	–
*P. volgarica* (PTV7)	[Russia] Saratovskaya Prov., Khvalynsky distr., near Novaya Yablonka vill., chalk hill slope over the Syzran-Saratov highway, I. Schanzer et al. No. SH200519-29, 20.05.2019, 52.372381° N, 47.967634° E (MHA)	A	MN849417	MN871393	–
*P. volgarica* (PTV8)	[Russia] Saratovskaya Prov., Khvalynsky distr., near Novaya Yablonka vill., chalk hill slope over the Syzran-Saratov highway, I. Schanzer et al. No. SH200519-30, 20.05.2019, 52.372381° N, 47.967634° E (MHA)	A	MN849418	MN871394	–
*P. volgarica* (PTV9)	[Russia] Saratovskaya Prov., Khvalynsky distr., near Novaya Yablonka vill., chalk hill slope over the Syzran-Saratov highway, I. Schanzer et al. No. SH200519-31, 20.05.2019, 52.372381° N, 47.967634° E (MHA)	A	MN849419	MN871395	–
